# Oxy-Inflammation in Humans during Underwater Activities

**DOI:** 10.3390/ijms25053060

**Published:** 2024-03-06

**Authors:** Alessandra Vezzoli, Simona Mrakic-Sposta, Andrea Brizzolari, Costantino Balestra, Enrico Maria Camporesi, Gerardo Bosco

**Affiliations:** 1 Institute of Clinical Physiology-National Research Council (CNR-IFC), 20142 Milano, Italy; alessandra.vezzoli@cnr.it; 2 Department of Biomedical Sciences, University of Padova, 35131 Padova, Italy; andreabrizzolari79@gmail.com (A.B.); gerardo.bosco@unipd.it (G.B.); 3 Environmental, Occupational, Aging (Integrative) Physiology Laboratory, Haute Ecole Bruxelles-Brabant (HE2B), 1160 Brussels, Belgium; cbalestra@he2b.be; 4 Physical Activity Teaching Unit, Motor Sciences Department, Université Libre de Bruxelles (ULB), 1050 Brussels, Belgium; 5 DAN Europe Research Division (Roseto-Brussels), 1160 Brussels, Belgium; 6 TEAM Health Anaesthesia, Tampa General Hospital, Tampa, FL 33606, USA; ecampore@usf.edu

**Keywords:** breath-hold diving, SCUBA, closed-circuit rebreather, saturation diving, oxidants, reactive oxygen species, reactive nitrogen species

## Abstract

Underwater activities are characterized by an imbalance between reactive oxygen/nitrogen species (RONS) and antioxidant mechanisms, which can be associated with an inflammatory response, depending on O_2_ availability. This review explores the oxidative stress mechanisms and related inflammation status (Oxy-Inflammation) in underwater activities such as breath-hold (BH) diving, Self-Contained Underwater Breathing Apparatus (SCUBA) and Closed-Circuit Rebreather (CCR) diving, and saturation diving. Divers are exposed to hypoxic and hyperoxic conditions, amplified by environmental conditions, hyperbaric pressure, cold water, different types of breathing gases, and air/non-air mixtures. The “diving response”, including physiological adaptation, cardiovascular stress, increased arterial blood pressure, peripheral vasoconstriction, altered blood gas values, and risk of bubble formation during decompression, are reported.

## 1. Introduction

SCUBA (Self-Contained Underwater Breathing Apparatus) and BH (breath-hold breathing) diving are breathtaking adventures that allow us to explore the world beneath the seas. However, diving comes with its own set of inherent risks and challenges. From time to time, thankfully rarely, SCUBA diving accidents or illnesses may occur. These can range from minor discomfort to life-threatening situations. One essential tool that plays a crucial role in mitigating the consequences of diving-related diseases is oxygen (O_2_) [1].

Regarding diving-related diseases, time is another vital parameter in diving activities. The inhalation of O_2_ (normobaric or hyperbaric, and with various types of masks, depending on whether the diver is conscious or unconscious) works after decompression sickness (DCS) by accelerating the diffusion and elimination of the excess nitrogen absorbed during SCUBA diving [2]. Diving-related activities are well known to produce partial O_2_ pressure (pO_2_) variations since the ambient pressure changes during underwater excursions; these changes can go from hyperoxia during hyperbaric phases and reach hypoxia on return from depths during some BH diving activities. Prolonged hyperoxia is known during SCUBA diving but does not always reach levels above 1 absolute atmosphere of O_2_ in the breathing mix [3]. Prolonged hyperoxia (apart from in medical settings) is not easy to reach under physiological conditions. However, it is still possible, for instance, if people are living below sea level around the “Dead Sea” in Israel (located 402 m below sea level, where the barometric pressure reaches 800 mmHg) [4]. The other setting to achieve a prolonged hyperoxia above 1 atmosphere of pure O_2_ breathing can be reached during Closed-Circuit Rebreather diving since the O_2_ set point for such dives is commonly set between 1.2 and 1.4 ATA during several hours of the excursion [5,6,7,8,9]. A chronic, less important form of hyperoxia is encountered during other saturation diving procedures where, depending on the methods used, around 0.49 ATA of partial O_2_ pressure (pO_2_) levels are kept during several days of decompression needed to reach sea level [10].

This review will discuss the impact of diving activities on oxidative/inflammatory stress (Oxy-Inflammation).

## 2. Oxy-Inflammation-Related Mechanisms and Outcomes

Humans and most terrestrial animal organisms have adapted to live in the Earth’s atmosphere, where the fraction of inspired oxygen (fiO_2_) equals approximately 0.21. O_2_ is the third most abundant element in the universe, after hydrogen (H_2_) and helium (He) as compounds, including oxides, which constitute around 50% of Earth’s crust [11]. Furthermore, O_2_ is one of the main components of biological macromolecules (proteins, carbohydrates, nucleic acids) and the last acceptor of electrons in oxidative phosphorylation, providing most of the natural energy in living organisms.

### 2.1. Oxidative Stress in Hypoxia and Hyperoxia

**Figure 1:**
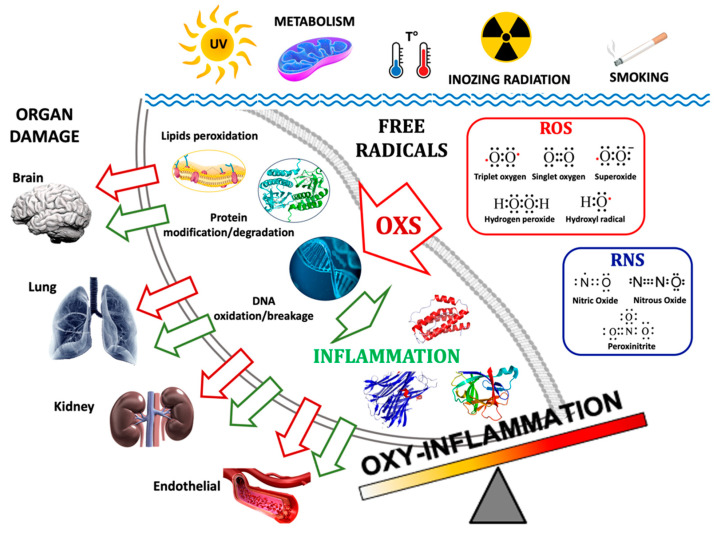
The main pathways of the formation of Oxy-Inflammation and organ damage. Environmental triggers such as exposure to UV and ionizing radiation, metabolism, water/body temperature, and smoking can produce free radicals: ROS (e.g., ^3^O_2_, O_2_, O_2_^−^, H_2_O_2_, HO·) and RNS (e.g., NO, N_2_O, ONOO^−^). Their accumulations can lead to lipids’ cellular membrane degradation, misfolded proteins, and DNA oxidation/breakage. The inflammation triggered by Oxi-Inflammation is the cause of many types of organ damage and acute/chronic diseases.

The redox system plays a crucial role in maintaining cell homeostasis and balancing the generation/elimination of reactive oxygen (ROS) and nitrogen (RNS) species [12]. A continuous flux of oxidant species guarantees redox homeostasis originated by mitochondria to deal with the electron consumption during the electron chain, resulting in the release of superoxide anions (O_2_^2−^) [13]. Being O_2_-derived compounds, ROS and RNS are “*double-faced*” elements because they carry out an essential function in several physiological mechanisms, including pathogen phagocytosis, modulating activities, and regulatory ability during the transduction of intercellular information [14]. On the other hand, an imbalance between ROS/RNS generation and detoxification leads to a condition of “oxidative stress” and the damage of biological macromolecules like lipids, proteins, and DNA [15] (see Figure 1). Following one of the most complete definitions, oxidative stress is “*the consequence of the failure to maintain the physiological redox steady state, which is the self-correcting physiological response to different challenges*” [16]. ROS such as O_2_^2−^ act both as a signaling molecule, contributing to the release of proinflammatory cytokine [17], and inflammatory mediator, reacting with nitric oxide (NO) to give peroxynitrite (ONOO^−^), an aggressive RNS that induces nitrosative stress, exacerbating the inflammation status [18]. Furthermore, the overproduction of ROS has been implicated in the development and progression of some diseases: cardiovascular, neurodegenerative, and metabolic diseases and cancer [19]. Elevated ROS levels contribute to stabilizing hypoxia-inducible factor-1α (HIF-1α), a key regulator of cellular responses to low oxygen. Additionally, ROS are essential messenger and signaling molecules in muscle adaptations to exercise and disuse, as documented by various studies [20]. The interplay between hypoxia-induced signaling pathways and oxidative stress is intricated [1,21].

On the other hand, an overdose of oxygen can harm cell membranes, lungs, and overall health by increasing oxidative stress and inflammation. For example, it damages the lungs, affecting surfactant proteins and mucociliary clearance, leading to lung collapse, reduced compliance, and higher infection risk [22]. Also, hyperoxemia constricts vessels, reducing coronary blood flow and cardiac output and potentially altering microvascular circulation [23].

### 2.2. Inflammation in Hypoxia and Hyperoxia

Inflammation may be influenced by fiO_2_ level, as reported by some authors investigating subjects exposed to hypoxia that showed pulmonary edema caused by inflammatory markers release, including interleukin-6 (IL-6) and protein C-reactive (CRP) [24,25,26]. To adapt body tissues to hypoxia, hypoxia-inducible factors (HIFs), a family of heterodimers that constitute a transcription complex that is stabilized by hypoxia [26] or a return from hyperoxia [27,28,29,30], are activated to coordinate several transcriptional pathways to optimize metabolic and vascular functions toward low fiO_2_ [31]. Furthermore, HIFs are controlled by prolyl hydroxylase domain (PHD) proteins, a class of “O_2_ sensors” that are involved in HIF level regulation and O_2_ homeostasis [32]. Nuclear factor κB (NF-κB) is involved in inflammation regulation and immune response [33], interacting with the PHD-HIF pathway [34], confirming the link between hypoxia and inflammation [35]. Other hypoxia–inflammation interactions involve the IκB kinase complex, an NF-κB regulatory component [31], and the regulation of HIF transcription before and during the inflammation [36]. Under inflammatory status, NF-κB activation stimulates immune defense by neutrophil release [37]. At the same time, HIF-α promotes type 2 helper T-cell (Th2) production [38] and macrophage activation with a ROS-dependent mechanism [39]. Inflammation onset against hypoxia can be clinically significant (e.g., chronic respiratory/renal/kidney/cardiovascular/neurological disease, mitochondrial/endothelial dysfunction, tumors) [21].

Hyperoxia represents the other side of the coin of fiO_2_ value. In this case, tissues are exposed to an excess supply of O_2_, with the arterial partial pressure of O_2_ (paO_2_) value being greater than 100 mmHg [40]. As mentioned above, exposure to prolonged hyperoxia exacerbates oxidative stress, leading to cellular damage via lipid peroxidation, enzyme inactivation, biological macromolecule oxidation, and apoptosis or necrosis [41]. Exposure to high fiO_2_ levels during significant time lapse results in O_2_ lung toxicity, characterized by a pulmonary inflammatory response [42] and a release of proinflammatory cytokines implicated in mediating neutrophil action into hyperoxic lungs [43] in the early stages of inflammatory response [44]. On the other hand, short-term hyperoxia seems to attenuate cytokine production [45], β2-integrin expression necessary for leukocyte adhesion [46], and macrophage phagocytosis and killing [47]. In animal models, some authors found that hyperoxia could mitigate the inflammatory response after glucan intake [48,49]. 

### 2.3. Endothelial Dysfunction

Increased partial O_2_ pressure (pO_2_) promotes oxidative stress, which triggers endothelial dysfunction. There are two mechanisms strongly related to the decrease in nitric oxide availability (NO). First, hyperoxia-related oxidative stress leads to the generation of the superoxide anion (O_2_^−^), which reacts with NO to produce peroxynitrite (ONOO^−^) [50]. ROS can also oxidize nitric oxide synthase (NOS) cofactor tetrahydrobiopterin (BH4) into dihydrobiopterin (BH2), which causes a reduced liberation of NO uncoupling endothelial NO synthase [51], leading to endothelial dysfunctions [52]. NO can be preserved by the activation of antioxidant defenses, as observed by several authors that found NO increases during the deep phases of both SCUBA and BH dives [53]. The same tendencies have been shown in SCUBA [54] or CCR diving [55]. After exiting saturation diving, endothelial recovery is fast, showing no long-term adverse effect on vascular function [56]. Furthermore, circulating bubbles may induce endothelial dysfunctions [57] through direct contact between microbubbles and endothelial cells [58] and the activation of a coagulation cascade [59,60]. However, we want to highlight that both deep and repetitive BH diving lead to endothelial dysfunction that may play an essential role in the genesis of neurological decompression sickness [61].

### 2.4. Hypoxia, Reoxygenation, and Hyperoxia

Redox balance/unbalance is significantly modulated by external factors such as environmental hypoxia [62,63,64,65,66,67,68], hyperoxia [69,70,71,72,73], and physical activity [74,75,76,77]. Moreover, both environmental hypoxia and physical activity have been shown to augment oxidative stress in a dose-dependent manner [77,78,79].

Reduced O_2_ tension during ischemia–reperfusion (IRI) episodes activates cellular pathways that upregulate proinflammatory signaling and promote oxidant generation [80]. Reperfusion after ischemia recruits inflammatory cells to the vascular wall, further exacerbating oxidant production and ultimately resulting in cell death, tissue injury, and organ dysfunction. As an IRI consequence, the activation of the NF-κB pathway leads to the release of proinflammatory cytokines (IL-1, IL-6, IL-8) [81] and major histocompatibility complex (MHC) upregulation [82]. Observing the correlation of the renal expression of Toll-like receptor (TLR) 4 with IRI degree [83], a link between IRI and innate/adaptive immunity has been hypothesized, as reported by some authors in transplant models [84,85]. 

Physical exercise has been proposed as a model of “intermittent hypoxia” (IH) or “intermittent hyperoxia” since oxidative stress increases during exercise, which can be defined as a combination of cycles of hypoxia and normoxia, hypercapnia, and hyperoxia, depending on the training type and duration [70,86,87]. Physical exercise, mainly moderate activity, improves health by reducing the risk of obesity and cardiovascular and chronic degenerative disease onset [88], raising NO availability, decreasing oxidative stress, and fostering antioxidant defenses [89]. On the other hand, exercise at altitude exposes tissue to hypoxia, which may increase oxidative stress by reducing antioxidant defense efficacy [90].

### 2.5. Oxy-Inflammation Biomarkers

The simultaneous measurement of a panel of biomarkers has been frequently chosen, as this can also provide a comprehensive assessment of oxidative stress effects on vascular function. Protein biomarkers were selected to cover critical features of vascular function, i.e., inflammation, endothelial function, and fibrinolysis. CRP is a biomarker of inflammation and endothelial function [91]; its increase is associated with impaired endothelium-dependent vasodilatation [92]. IL-6 increases the adhesiveness of the endothelial cells for lymphocytes by upregulating Intercellular Adhesion Molecule-1 (ICAM-1) for a proinflammatory reaction [93].

In this review, we use the term “Oxy-Inflammation”, proposed by Valacchi et al. [94], to describe “*a condition characterized by a combination of systemic oxidative stress associated with an inflammatory condition*”. This review summarizes the “Oxy-Inflammation” in underwater activities such as breath-hold (BH) and SCUBA diving.

The main mechanisms of the formation of Oxy-Inflammation and relative organ damage are illustrated in Figure 1.

## 3. The Oxy-Inflammation Mechanism in Underwater Activity

### 3.1. Breath-Hold (BH) Diving

BH diving elicits a physiological adaptation collectively termed the “diving response”, which includes changes such as bradycardia, reduced cardiac output, increased arterial blood pressure, peripheral vasoconstriction, and altered blood gas values [95]. Hyperoxia occurs during descent and bottom time in BH divers due to increased environmental pressure and thorax compression [96,97]. Hypoxia-induced endothelial damage might be less pronounced because hypoxia appears in the last portion of the ascent phase of a dive [96,97,98,99].

Hyperoxia-induced changes observed in BH diving may influence arterial blood gas (ABG) parameters, especially PaO_2_ and arterial pCO_2_ (PaCO_2_). At the maximum depth, PaO_2_ increases due to hyperoxia-induced effects caused by the rise in environmental pressure [99,100,101,102], but this trend was not observed in all BH divers. This unexpected PaO_2_ decrease is related to a lung volume reduction leading to a right-to-left shunt through lung atelectasis [101,102,103,104]. Furthermore, Bosco et al. [105] reported that BH diving-related exercise breaks down PaO_2_ levels due to muscle O_2_ consumption [105], which may increase the risk of injuries [106] during the ascent phase. PaCO_2_ values do not change much, probably due to the body’s capacity to store soluble CO_2_ [101] and hyperventilation that decreases PaO_2_ [98].

Similarly to PaO_2_, PaCO_2_ variation may be related to BH diving type, as reported by Bosco et al., who found higher PaCO_2_ values after static apnea concerning those after a deep dive [105], which reduced from initial values. In the case of deep diving, PaCO_2_ rises in BH divers who do muscular work when returning to the surface using fins. The muscular work contribution may be confirmed by the higher augmented lactate value that was not recorded in other conditions [105].

In BH diving, IH can be a powerful trigger for endothelial dysfunction [106,107]. More severe diving stress in repetitive BH dives could lead to strains on the cardiovascular system, vascular function, and endothelial integrity. Endothelial dysfunctions in BH diving have been investigated by Theunissen et al. [54], where the reduction in NO-dependent vascular function resulted from low NO bioavailability. The same authors also showed a decrease in this endothelial dysfunction when supplementing antioxidants to the divers [108].

High pO_2_ may also promote oxidative stress due to the hyperbaric environment, increasing free O_2_ that may generate ROS [109]. Oxidative stress has been investigated in BH diving [54,107,108,109], confirming its role in the development of endothelial dysfunction [110]. The overproduction of ROS and consequent oxidative damage to membrane lipids and total antioxidant capacity (TAC) reflects a hypoxic condition in the last few meters while ascending to the surface [109]. Neopterin and creatinine levels increase, which suggests an alteration in renal function as a physiological response to pO_2_ variations during the dive [109,110].

Changes in polyunsaturated fatty acids (PUFAs) and eicosanoids after a single apnea event revealed different kinetics of pro- and anti-inflammatory regulations and changes in oxidative stress levels [111].

Adaptation to hypoxia may involve amino acid metabolism [112,113], and hypoxia might be a stimulus for amino acids’ release in the bloodstream because serine and taurine may be engaged in hypoxia adaptation, especially during the ascent phase of a BH dive [113].

Furthermore, prolonged exposure to hypoxia, such as diving at high altitudes, may affect platelet activity, as reported by some authors [114], but this aspect seems to be related to pO_2_ [115]. Platelet activation can be induced by exercise-related inflammation [116] engaging with leukocytes via soluble factors and physical interaction with several receptors, including platelet P-selectin (CD62P), CD40 ligand (CD40L), as well as PSGL-1, CD40, and Mac-1 (integrin αMβ2, CD11b/CD18) on leukocytes [117,118]. Platelet–leukocyte interactions facilitate leukocyte recruitment and extravasation to inflammation sites, leading to leukocyte proinflammatory mediator release but, in some cases, damping inflammation [119,120]. Furthermore, some authors found a link between gene activity and immune cell activation against inflammation, observing a temporary increase in leucocytes and an upregulation of the anti-inflammatory response genes [121].

As reported by Wang et al. [122], ultrasound lung comets (ULCs) may be associated with lung inflammation and represent a tool to evaluate heart alteration [122], and some mechanical pressure changes in the chest provoked diaphragmatic spasms with a closed glottis (during the struggling phase of breath-holding), leading to lung alveolo-vascular stress [123]. Paganini et al. [101] investigated ULC during a BH dive at increasing depths, observing the onset of lung comets in some subjects; this may have been due to an altered recovery from atelectasis [103] that seemed to have been caused by the alternating closing and opening of bronchioles and alveoli during the dives [102]. As reported by Tojo et al. [124], atelectasis may lead to alveolar hypoxia-induced inflammation, releasing NF-κB-dependent CXCL-1 from epithelial cells and activating HIF-1α toward alveolar hypoxia [124]. 

Interestingly, cetaceans exhibit physiological adaptations that allow the transition to aquatic life, including a robust antioxidant defense system that prevents injury from repeated exposure to ischemia–reperfusion events associated with BH diving. x. Heme oxygenase (HO) activity increased in dolphin cells but not human cells. On the contrary, TNF-α expression increased in human cells but not in dolphin cells [125]. 

HO is a cytoprotective protein with anti-inflammatory properties, catalyzing the first step in the oxidative degradation of heme. Various stimuli, including hypoxia, oxidant stress, and inflammatory cytokines, regulate the inducible HO-1 isoform.

Some authors investigated microparticle (MP) release after a BH-diving training protocol, observing that the alterations in endothelial reactivity were coupled to increased levels of Endothelial MPs (EMPs) (CD31+/41−), markers of endothelial apoptosis [126,127]. Intermittent hypoxia could increase cellular stress [61]. An increase in CD31+/annexin+EMP level was observed in some volunteers exposed to normobaric hypoxia in a chamber with oxygen saturation (SaO_2_) reduced to 73% ± 10% [3]. A similar increase in CD144 EMPs was found in some BH divers who performed single maximal dry BH (SaO_2_ of 72% ± 12%). Both experiments were conducted in dry conditions but showed that short-term hypoxic situations could trigger endothelial cell stress. Nevertheless, diving stress in BH diving is only partly related to hypoxia [127]. 

**Figure 2:**
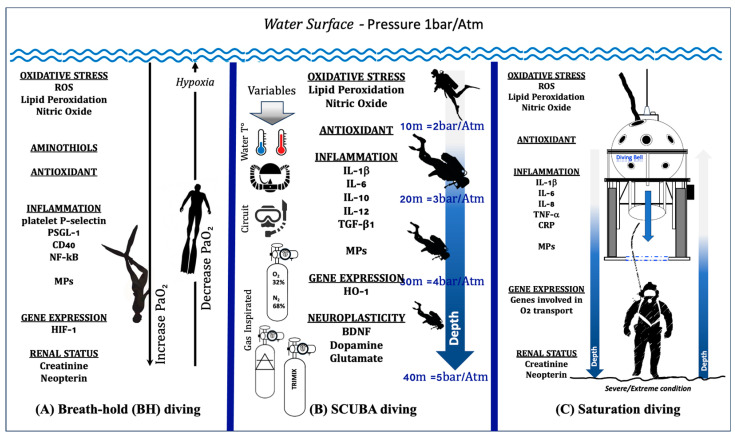
Illustration of up–down regulation biomarkers in different human underwater activities: (**A**) Breath-hold diving. The physiological changes in PaO_2_ during the descent and ascent of a deep breath-hold dive are one of the causes of biological changes at a systemic and molecular level. (**B**) SCUBA diving. The response in SCUBA diving varies depending on number of dives carried out, depth, water temperature, type of breathing circuit (open vs. CCR), gases inspired, and exercise. (**C**) Saturation diving. In the sketch, the saturation worksite is shown. The working depth corresponds to the maximum depth reached by the divers, where the “scenario” is considered a severe/extreme condition. All these activities involve changes in levels of Oxy-Inflammation, redox states (aminothiols), gene expression, antioxidants, markers of neuroplasticity, and renal status.

In Figure 2A, we illustrate up–down regulation biomarkers in breath-hold diving.

### 3.2. Self-Contained Underwater Breathing Apparatus (SCUBA) and Closed-Circuit Rebreather (CCR) Diving

One of the most commonly known activities that leads to hyperoxia is SCUBA diving. SCUBA diving exposes the human body to environmental stress represented by increased pO_2_, physical effort, and elevated breathing resistance, which alter the endothelial function [128,129,130]. SCUBA diving-related physical activity can induce oxidative and cardiovascular stress, amplified by environmental conditions, including hyperoxia, hyperbaric pressure, and cold water [131,132].

The mechanisms that lead to endothelial dysfunction in SCUBA diving may result from increased hyperoxia-induced oxidative stress and peripheral vasoconstriction [129]. The hyperoxia associated with SCUBA diving results in ROS production, which activates the antioxidant defenses, leading to an increased expression of several antioxidant enzymes, such as catalase (CAT) [133], glutathione peroxidase (GPx) [134], and superoxide dismutase (SOD) [135]. As CAT activity rises in lymphocytes within a few hours after the dive, while GPx activity rises after surfacing, ROS production stimulates lymphocyte mobilization because GPx is one of the first antioxidants activated to detoxify ROS [134]. SOD activity increases to protect against ONOO^−^ and to preserve NO generation [135]. Due to the mobilization of endogenous antioxidant systems, the total antioxidant capacity increases after surfacing to help control vascular oxidative stress, activating a signaling cascade that stimulates resistance against diving-related oxidative stress [136]. 

Oxidative stress may also impair cognitive functions during a dive due to brain-derived neurotrophic factor (BDNF) loss, followed by a decrease in dopamine and glutamate, especially during decompression [137]. This BDNF deficit seems to alter excitatory/inhibitory neurotransmission, including GABA receptors [138], whose interaction with N_2_ has been proposed as a mechanism of nitrogen narcosis [137,138,139,140,141,142,143].

Pressure changes during a dive or decompression significantly influence the hematocrit component level, including leukocytes and platelets [144,145]. Some authors observed that SCUBA diving did not affect the number of lymphocytes but their functions were affected, while no changes in H_2_O_2_ production in lymphocytes and GPx were found. Additionally, CAT activities increased [135]. On the other hand, Morabito et al. [146] showed significantly decreased levels of H_2_O_2_ in lymphocytes, probably due to increased CAT activity and endogenous antioxidant enzymes. 

Dumić et al. [147] reported that regular recreational SCUBA diving promotes an anti-inflammatory status, thus contributing to cardioprotection and conferring multiple health benefits. Divers who performed five dives, one per week, at a depth of 20–30 m that lasted 30 min, manifested IgG and TPP N-glycosylation alterations toward anti-inflammatory status over the whole study period with an increase in monogalyctosylated and core-fucosylated IgG N-glycans and a decrease in galactosylated TPP N-glycans. On the other hand, military combat swimmers (O_2_ divers) are regularly exposed to hyperbaric hyperoxia in addition to intensive endurance training intervals and, therefore, are exposed to extreme levels of oxidative stress. Compared to controls, they exhibited a proinflammatory immune status exemplified by an elevated number of CD4+CD25+ T cells, the elevated expression of proinflammatory cytokine IL-12, and the diminished expression of anti-inflammatory TGF-β1 [148]. Supported by decreased basal gene expression and the prolonged upregulation of anti-oxidative HO-1, these data suggest that higher oxidative stress levels, as those present under intermitted hyperbaric hyperoxia conditions, e.g., through oxygen diving, promote a higher inflammatory immune status.

Breathing air in hyperbaric condition leads to the formation of venous gas emboli (VGE) that interact with the endothelium [149], leading to platelet aggregation to the bubble surface [150] and resulting in the formation of microthrombi in lung vessels after decompression in the DCS model [151,152].

In addition to a mechanical explanation for DCS, an inflammatory mechanism has been suggested [153]. In an observational study carried out on a similarly well-matched control group consisting of equally fit non-divers with identical physical training and living conditions before and after a 2-month period of daily diving, no changes in IL-6 and IL-1 receptor antagonist (IL-1ra), but an increase in IL-8 and neutrophil gelatinase-associated lipocalin (NGAL), together with a decrease in secretory leukocyte protease inhibitor (SLPI), were found [154]. The findings suggest inflammatory activation that is not severe because no changes in IL-6 or IL-1ra were found. The increase in NGAL and IL-8 levels was interpreted as a sign of leukocyte activation. The decreased SLPI levels suggest an influence on the inflammatory defense mechanism. Therefore, the compensated activation of the inflammatory defense mechanism without the loss of homeostasis of the inflammatory system was shown. The formation of bubbles during decompression may trigger this inflammatory response. It is also probable that the interplay between pro- and anti-inflammatory substances is a fine-tuned balancing act and that it is only when this balance collapses that morbidity ensues.

Different types of breathing gas are used in SCUBA diving: air and non-air mixtures [155]. The latter contains mainly oxygen, nitrogen, and helium that form nitrox (nitrogen + oxygen), heliox (helium + oxygen), and trimix (oxygen, helium, and nitrogen) with different advantages and features [9,155]. Nitrox is mainly used for shallow recreational dives, heliox for deep diving, and trimix for deep but short dives to avoid neurological side effects. The use of trimix in SCUBA diving has recently become more widespread to reduce nitrogen narcosis compared to air diving [140,141,142].

Some experimental studies, although conflicting, invoked a possible anti-inflammatory role of helium [156]. This gas could increase NO, induce nuclear factor erythroid 2-related factor 2 activation, and cause consequent reductions in systemic inflammation and oxidative stress. Recently, increases in extracellular vesicles and inflammatory status (IL-1) were found in CCR SCUBA divers, highlighting a decrease in plasma gelsolin (pGSN) [157]. It has been demonstrated that pGSN modulates the production of IL-1β-containing microparticles following high-pressure exposure and decompression [158].

Another study [159] found no difference in most of the inflammatory factors (including monocyte chemoattractant protein 1) measured between the trimix and air groups, casting doubt on the protective effect of helium. Following the dive, IL-6 values slightly increased, while IL-8 and epithelial growth factor (EGF) decreased in both groups without significant variation.

To reduce bubble formation and platelet activation, some authors proposed a hyperbaric pretreatment with O_2_ before the dive, observing a significant decrease in decompression-induced bubbles [160,161] and the activation of antioxidant enzymes such as CAT and SOD [162]. The increased O_2_ diffuses in micronuclei in substitution of N_2_, which is eliminated through the lungs and then absorbed, reducing micronuclei formation [163]. Furthermore, the reduction in pN_2_ and breathing enriched air nitrox (EAN) can reduce platelet activation [164], as well as breathing normobaric O_2_ before a dive [115]. On the other hand, exposure to EAN mixtures and diving-related physical activity can raise ROS generation [129]; oxidative stress biomarkers including 8-isoprostane and 8-deoxyguanosine; and inflammatory markers such as IL-1β, IL-6, and TNFα [165]. 

Bosco et al. [165] observed oxidative stress markers’ increase after a dive using close-circuit rebreather (CCR) apparatus, probably originating from increased fiO_2_, amplified by additional variables such as physical exercise [166] and breathing resistance [167]. On the other hand, mild exercise during a dive may be enough to allow VGE transit, suggesting a potential benefit of exercise before a dive [168].

No association between the decompression model chosen for a 50 msw dive and the likelihood of DCS was established [169]. The dive profiles induced only minor changes in inflammatory markers. The significant increase in IL-6 and decreased IL-8 and EGF levels observed in groups following the ratio decompression strategy (RDS) or compartmental decompression models (CDMs) were within the normal range and were consistent with exercise-induced changes. However, compared to CDM, the RDS-controlled model has the disadvantage of the increased secretion of chemokines in developing vascular damage. This increased secretion of proinflammatory chemokines seems related to the decompression system rather than the more prolonged exposure to high partial pressures of oxygen that RDS divers undergo.

A short-term ketogenic diet has been observed to be effective in weight loss, decreasing inflammation, and protecting against lipid peroxidation during a dive [165]. 

In Figure 2B, we illustrate up–down regulation biomarkers in SCUBA diving.

### 3.3. Saturation Diving

A particular case of diving activity is “saturation diving”, which describes a diving method that enables the diver to stay under pressure for a long time (several hours or even days), practiced by military forces and commercial divers. Saturation diving is an established way to conduct subsea operations with human intervention. Saturation divers are exposed to severe/extreme environmental conditions, exposing themselves to higher risks and accidents [170,171,172,173,174,175]. 

Saturation diving involves divers staying under pressure until their tissues are saturated with breathing gas, exposing them to increased oxygen pressure, potential toxic gases, and the risk of bubble formation during decompression. Prolonged exposure to hyperoxia can generate ROS, damaging cell structures like lipids, proteins, and nucleic acids. All these factors, coupled with vascular gas bubble formation, may lead to endothelial dysfunction. The diver’s antioxidant status, influenced by diet and genetics, plays a crucial role in protecting against injuries caused by these conditions [174].

While working, divers must acclimatize to hyperbaric environments [175,176,177]. In saturation diving, hyperoxia, partial gas pressure changes, and inert gas exchange during decompression are likely sources of excess ROS [178]. Mrakic-Sposta et al. [178] found an increase in oxidative stress-related biomarkers, including 8-deoxyguanosine, 3-nitrotyrosine, and neopterin, due to prolonged exposure to higher pO_2_ value. Furthermore, saturation divers showed elevated concentrations of IL-6, the first cytokine released into the circulation during inflammation response [10,178,179,180,181,182], and creatinine in the post-dive stage, reflecting the systemic effects induced by hyperoxia, hyperbaric pressure, and exercise at depth [150]. 

Moreover, Krog et al. [183] demonstrated the increase in Natural Killer (NK) cell cytotoxicity during the compression phase accompanied by alterations in the cytotoxic NK cell subsets (CD32CD16brightCD56dim) in saturation divers’ peripheral blood, with a reduction in glutathione levels [184,185].

Previous studies have concluded that high oxidative stress in divers may be reversed through antioxidant vitamin supplementation [186,187]. In an earlier experiment, the supplementation of vitamin C, α-tocopherol (vitamin E), and tea catechins given to divers during a 400 msw deep saturation dive every day for 40 days prevented hepatic disturbances [188]. A more recent publication demonstrated that divers’ endogenous antioxidant mechanisms counteracted the effects of hyperbaric hyperoxia after 200 msw saturation diving [184]. Sparse data showing the impact of antioxidant vitamins on vascular function in diving are available; however, Deb et al.’s [189] review on antioxidant effects in saturation diving subjects indicates that care is warranted when choosing whether and how to give antioxidant supplements [190].

The iron reserve can also be a valid aid in saturation diving. Data provided by Zwart et al. [179] showed that elevated body iron stores in a hyperoxic environment trigger a DNA damage repair response in peripheral blood mononuclear cells, excluding double-stranded DNA damage. Furthermore, folate levels decrease rapidly in this setting, suggesting higher folate requirements may be necessary when both body iron stores and DNA damage repair responses are elevated. This observation indicates potential interplay between iron status, O_2_ levels, and folate in cellular responses.

Acclimatization to professional saturation diving was associated with post-saturation gene expression changes to elevated O_2_ partial pressure by the extensive downregulation of factors involved in O_2_ transport, including heme, hemoglobin, and erythrocytes. Primary endogenous antioxidants, such as SOD-1, CAT, and glutathione synthetase, were upregulated, and there was increased expression of genes involved in immune activity and inflammatory signaling pathways [186].

The observation that a hyperbaric and hyperoxic environment in saturation diving induces proinflammatory responses that help the body adapt to the inherent oxidative stress in maintaining homeostasis was confirmed recently by Monnoyer et al. [191]. They reported elevated salivary levels of CRP, IL-1β, IL-8, SIgA, and TNF-α during the bottom phase of the hyperbaric saturation, whereas IL-6, cortisol, and alpha-amylase were unchanged. All changes observed during saturation were abolished at the end of decompression. Figure 2C illustrates up–down regulation biomarkers in saturation diving.

## 4. Conclusions

Several studies encompassed the possibility of mitigating some diving-related impairments; in fact, underwater activities induce oxidative stress and inflammatory-related parameter changes depending on O_2_ availability. While in SCUBA diving, these changes are related only to hyperoxia onset, in BH diving, they are a consequence of hyperoxia/hypoxia alternation. As described, the presence of ROS, RNS, and inflammation can result in organ dysfunction (e.g., endothelium, lung, kidney, brain) with complex interplay activity [192,193,194,195,196]. In the recreational field, several preconditioning methods have been considered which have challenged previously accepted ideas [197,198,199,200]. One direction was chosen to mitigate diving-related risks: one aiming to endothelial dysfunction, although conduit arteries and microcirculation seem not to behave in parallel [201,202], and supplementation with antioxidant agents before diving [203,204,205,206,207].

Future research should compare and correlate different environmental conditions (e.g., hot/cold water, sea/lake/swimming pool) in different underwater activity scenarios from BH to SCUBA diving. Furthermore, it should also consider the dose-dependent effects of the gas/mixture alongside dive time and depth, associated with decompression times and with the effects of changes in oxygen concentrations. To date, the risk/benefit ratio has not yet been clearly defined. 

## Data Availability

Not applicable.

## Abbreviations

AA	Amino acid
ABG	Arterial blood gas
BH	Breath-hold diving
CAT	Catalase
CCR	Closed-Circuit Rebreather
CDM	Compartmental decompression model
CRP	Protein C-reactive
DCS	Decompression sickness
EGF	Epithelial growth factor
HIF	Hypoxia-inducible factor
IL-	Interleukin
IRI	Ischemia–reperfusion
NO	Nitric oxide
NK	Natural Killer
O_2_	Oxygen
O_2_^2−^	Superoxide anion
ONOO	Peroxinitrite
OxS	Oxidative stress
PaCO_2_	Arterial partial CO_2_ pressure
pO_2_	Partial O_2_ pressure
pGSN	Plasma gelsolin
RDS	Ratio decompression strategy
ROS	Reactive oxygen species
RNS	Reactive nitrogen species
SCUBA	Self-Contained Underwater Breathing Apparatus
SOD	Superoxide dismutase
TAC	Total Antioxidant Capacity
8-OH-dG	8-OH-2-deoxyguanosine
8-iso-PGF2α	8-isoprostane
